# The Discovery of Novel BCR-ABL Tyrosine Kinase Inhibitors Using a Pharmacophore Modeling and Virtual Screening Approach

**DOI:** 10.3389/fcell.2021.649434

**Published:** 2021-03-04

**Authors:** Ting-Ting Huang, Xin Wang, Shao-Jia Qiang, Zhen-Nan Zhao, Zhuo-Xun Wu, Charles R. Ashby, Jia-Zhong Li, Zhe-Sheng Chen

**Affiliations:** ^1^School of Pharmacy, Lanzhou University, Lanzhou, China; ^2^College of Pharmacy and Health Sciences, St. John’s University, Queens, NY, United States

**Keywords:** CML, BCR-ABL, pharmacophore model, ZINC21710815, apoptosis, autophagy

## Abstract

Chronic myelogenous leukemia (CML) typically results from a reciprocal translocation between chromosomes 9 and 22 to produce the *bcr-abl* oncogene that when translated, yields the p210 BCR-ABL protein in more than 90% of all CML patients. This protein has constitutive tyrosine kinase activity that activates numerous downstream pathways that ultimately produces uncontrolled myeloid proliferation. Although the use of the BCR-ABL tyrosine kinase inhibitors (TKIs), such as imatinib, nilotinib, dasatinib, bosutinib, and ponatinib have increased the overall survival of CML patients, their use is limited by drug resistance and severe adverse effects. Therefore, there is the need to develop novel compounds that can overcome these problems that limit the use of these drugs. Therefore, in this study, we sought to find novel compounds using Hypogen and Hiphip pharmacophore models based on the structures of clinically approved BCR-ABL TKIs. We also used optimal pharmacophore models such as three-dimensional queries to screen the ZINC database to search for potential BCR-ABL inhibitors. The hit compounds were further screened using Lipinski’s rule of five, ADMET and molecular docking, and the efficacy of the hit compounds was evaluated. Our *in vitro* results indicated that compound ZINC21710815 significantly inhibited the proliferation of K562, BaF3/WT, and BaF3/T315I leukemia cells by inducing cell cycle arrest. The compound ZINC21710815 decreased the expression of p-BCR-ABL, STAT5, and Crkl and produced apoptosis and autophagy. Our results suggest that ZINC21710815 may be a potential BCR-ABL inhibitor that should undergo *in vivo* evaluation.

## Introduction

Chronic myelogenous leukemia (CML) is a hematopoietic neoplastic disease that primarily results from the reciprocal translocation between chromosomes 9 and 22 (t9;22) (q34; q11) in a hematopoietic stem cell (HSC), resulting in the formation of the *bcr-abl* oncogene (Deininger et al., 2005; Ren, 2005; Kimura, 2006). Typically, the most common BCR-ABL proteins produced in CML patients, based on the loci of the chromosomal breaks, are p210, p190, and p230 (Winter et al., 1999; Arana-Trejo et al., 2002). The p210 protein occurs was observed in more than 90% of CML patients and has been hypothesized to be the initial step in the pathogenesis of CML (Arana-Trejo et al., 2002). The p210 protein is a constitutively active, non-receptor tyrosine kinase that phosphorylates its Tyr177 residue, which subsequently activates a number of downstream signaling pathways that causes HSC cells undergo significant abnormal proliferation and differentiation compared to normal cells, producing the pathogenic changes reported in CML patients (Deininger et al., 2000; Gora-Tybor and Robak, 2008; Cao et al., 2015; Wang et al., 2016).

Imatinib (Gleevec), which selectively targets the ATP binding site of the BCR-ABL protein, is a first generation BCR-ABL tyrosine kinase inhibitor (TKI) that was approved in 2001 by the FDA for treating CML (Gontarewicz et al., 2008; Wu et al., 2014). Initially, it was reported that imatinib significantly increased the survival time of CML patients (Machova Polakova et al., 2015; Deng et al., 2020). However, subsequent clinical trials indicated that patients with advanced-stage CML experience relapse due to development of resistance caused by point mutations within the BCR-ABL domain (notably T315I), amplification of the BCR-ABL protein, overexpression of BCR-ABL protein or the ABC transporter, *P*-glycoprotein (Coutre et al., 2000; von Bubnoff et al., 2002; Apperley, 2007; Tanaka and Kimura, 2008).

The second generation TKIs, dasatinib, nilotinib, and bosutinib, have been shown to have efficacy in treating CML patients with certain BCR-ABL mutations, but not those with the T315I mutation (O’Hare et al., 2005; Abbas and Hsyu, 2016). The mutations of BCR-ABL are a recognized cause of resistance to second-generation, especially T315I (Baccarani et al., 2009; Khorashad et al., 2013). Ponatinib, a third-generation TKI, is also efficacious in a number of mutations in the BCR-ABL protein, including T315I (Okabe et al., 2014). Compound mutations of BCR-ABL (T315I/F359V or Y253H/T315I) are usually resistant against ponatinib therapy (Zabriskie et al., 2014). However, ponatinib has been reported to significant adverse cardiovascular effects, notably serious venous thromboembolic events (Bu et al., 2014). Therefore, there is still the need to develop highly efficacious and tolerable BCR-ABL inhibitors for the treatment of CML.

Hypogen pharmacophore model were built based on known inhibitors, the structures and activity data of known active compounds were analyzed to generate quantitative 3D pharmacophore models with common pharmacodynamic action models of the compounds, which can be used to predict the efficacy of unknown compounds (Rampogu et al., 2018; Musoev et al., 2019). The Hiphop pharmacophore models were qualitative pharmacophore models generated according to the common characteristics of known inhibitors without considering the activity data of compounds (Zheng et al., 2009). Pharmacophore models can provide guidances for drug design and virtual screening.

In this study, the quantitative Hypogen pharmacophore model and the qualitative Hiphop model were built, these models were then used to screen the ZINC database to identify potential BCR-ABL inhibitors. The identified hit compounds were further evaluated using Lipinski’s rule of five, ADMET and CDOCKER docking. The molecules obtained from the Hypogen and Hiphop pharmacophore models mapping were selected to determine their anti-proliferative efficacy in specific leukemia cell lines. Subsequently, the skeleton of the most active compound was chosen to identify additional derivatives from the database to perform structural optimization. Finally, our results led us to evaluate the efficacy of the compound ZINC217108151 to: (1) inhibit the proliferation of certain leukemia cell lines; (2) inhibit the phosphorylation of the p210 BCR-ABL tyrosine; and (3) affect the activity of the downstream proteins, STAT5 and Crkl, which are activated by BCR-ABL.

## Materials and Methods

### Virtual Screening

**FIGURE 1:**
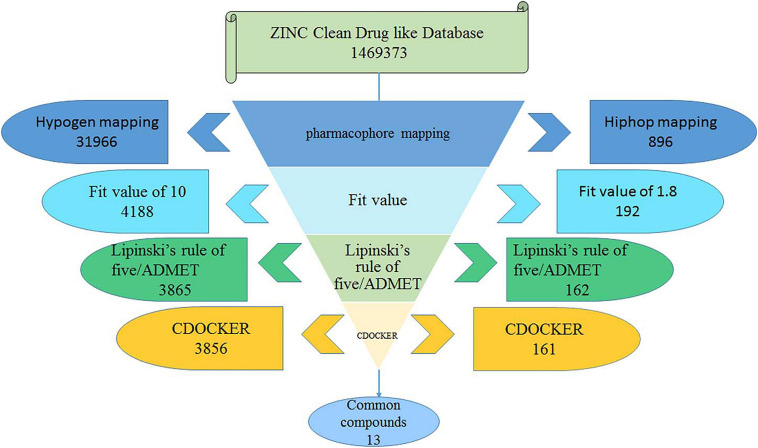
The flow chart for the virtual screening process.

The best Hypogen and Hiphop pharmacophore models (the method of building pharmacophore shown in Supplementary Material) were used as 3D structures to search the ZINC clean drug–like database (1,469,373 molecules) to obtain the compounds that matched the chemical features (Bhadauriya et al., 2013; Kumar et al., 2015). The workflow of each screening step and the number of compounds is shown in detail in Figure 1. Compounds that satisfied the fit value of Hypogen and Hiphop were retained. All hit compounds were evaluated using Lipinski’s rules of five and absorption, distribution, metabolism, excretion, and toxicity (ADMET) screening process (Rampogu et al., 2018). Lipinski’s rule of five was predicted by using calculate molecular properties in small molecules protocol in DS2.5. Lipinski’s rule of five consists of molecular weight ≤500 Daltons, rotatable bonds number ≤10, hydrogen bond acceptors ≤10, and hydrogen bond donors ≤5 (Rampogu et al., 2017). ADMET was predicted by using ADMET descriptors in calculate molecular properties protocol in DS2.5. The cut-off value of the solubility, absorption, and the BBB were 3, 0, and 3, respectively (Rampogu et al., 2018). Finally, the database was further screened using CDOCKER. Docking analysis was used to determine whether small molecules can enter the active site of the protein (Rampogu et al., 2020). The three-dimensional structure of ABL tyrosine kinase receptor (PDB: 1IEP) was obtained from Protein Data Bank (Protein Data Bank, 1971; Machova Polakova et al., 2015). All ligands were minimized by Chemistry at Harvard Macromolecular Mechanics (CHARMm) force field and the protein was prepared by removing all of the water molecules, adding hydrogen atoms and filling the loop area (Al-Balas et al., 2013). The active site was defined as 12.9 Å around the co-crystal ligand. The prepared protein and ligands were imported to perform CDOCKER docking. Higher docking energy and interaction energy represent the favorable binding between the protein and the ligands (Rampogu et al., 2018).

### Reagents and Cell Culture

After virtual screening, the most optimal compounds were purchased from TargetMol company (Boston, MA, United States) and dissolved in DMSO to make a stock solution (1 × 10^–1^ mol/L) and stored at −20°C for future use.

K562, BaF3/WT, and BaF3/T315I leukemia cells lines were grown in RPMI-1640 medium supplemented with 10% fetal bovine serum (Sijiqing, China) and 1% penicillin and streptomycin. Normal human embryonic liver diploid cells (CCC-HEL-1) were grown in Dulbecco’s modified Eagle’s medium (DMEM, HyClone) supplemented with 10% fetal bovine serum (GIBCO, Australia) and 1% penicillin and streptomycin, all cells were maintained in humidified air at 37°C with 5% CO_2_ (Gupta et al., 2020).

Antibodies C-Abl and p-C-Abl were purchased from Cell Signaling Technology (MA, United States), p-Crkl was purchased from Affinity Biosciences (OH, United States), p-STAT5A and β-actin were purchased from Bioss (MA, United States), LC3 was purchased from Sigma-Aldrich (United States), Beclin1 was purchased from Abcam (Cambridge, United Kingdom), Caspase3 was purchased from Cell Signaling Technology (MA, United States). HRP-conjugated Affinipure Goat Anti-Rabbit antibody was purchased from Proteintech (IL, United States).

### 3-(4,5-dimethylthiazol-2-yl)-2,5-diphenyltetrazolium Assay

K562, BaF3/WT, and BaF3/T315I leukemia cells were seeded in 96-well plates at a density of 5 × 10^3^ cells/well and 50 μL of various drug concentrations were added. CCC-HEL-1 cells were grown in 96-well plates at the same density and exposed to the same drug concentrations. The plates were incubated at 37°C for 72 h. Cell proliferation was evaluated using MTT [3-(4,5-dimethylthiazol-2-yl)-2,5-diphenyltetrazolium] assay. Fifteen μL of the MTT solution was added to each well of 96-well and plates were continually incubated at 37°C for 4 h and three liquid solutions. SDS, HCl, and isobutanol were added to each well and the plates were incubated at 37°C overnight. The optical density (OD) of each sample was measured at a wavelength of 570 nm and the 50% inhibitory concentration (IC_50_) was determined by from the concentration–response curve.

### Cell Cycle and Cell Apoptosis Analysis

K562 cells were plated in 6-well plates with 1 × 10^6^ cells/well, BaF3/WT and BaF3/T315I cells were plated in 6-well plates with 5 × 10^5^ cells/well. After all of the cells were incubated with 0 (vehicle), 0.1, 1, or 10 μM concentration of the test compounds for 24 h, they were harvested and washed with PBS and 1 ml of 70% cold ethanol was added to the cells (Kim et al., 2015; Jiang et al., 2016). The ethanol-fixed cells were stored at −20°C overnight. Subsequently, the cells were washed with PBS and centrifuged at 1,800 rpm for 8 min to obtain the pellet. The pellet was resuspended in 100 μL of RNase A, incubated at 37°C for 30 min, and mixed with 400 μL of propidium iodide (PI) and incubated at 4°C in the dark for 30 min. DNA content was determined using flow cytometry, at an emission wavelength of 488 nm and analyzed using ModFit LT software.

All cells were plated in 6-well plates with 5 × 10^5^ cells/well and incubated with 0 (vehicle), 0.1, 1, or 10 μM of the test compounds for 24 h. The cells were harvested, washed twice with cold PBS, resuspended with binding buffer, stained using Annexin V- fluorescein isothiocyante (FITC) and PI and mixed with PBS to obtain a final volume of 500 μL. Cellular apoptosis was determined using flow cytometry method within 1 h of staining and dot plots were set up to detect FITC and PI. Cells stained with FITC and without PI (FITC+, PI-) were categorized as being in early apoptosis, and cells stained with both dyes (FITC+, PI+) were categorized as being in late apoptosis or necrotic (Peng et al., 2001).

### Western Blot Analysis

Cells were plated in 6-well plates with 5 × 10^5^ cells/well and incubated with 0 (vehicle), 0.1, or 1 μM of the test compounds for 48 h. The harvested cells were washed with PBS, and lysed in a RIPA lysis buffer and collected by centrifugation at 14,000 × *g* for 15 min at 4°C. The cell extracts were separated by SDS-polyacrylamide gel electrophoresis (PAGE) and transferred to PVDF membranes. The membranes were blocked with 5% non-fat milk at room temperature for 75 min. The membranes were incubated with C-Abl, p-C-Abl, p-Crkl, p-STAT5A, β-actin, LC3, Beclin1 or caspase-3 antibodies overnight at 4°C, then washed three times with TBST and incubated for 1 h with the HRP-conjugated Affinipure Goat Anti-Rabbit antibody at room temperature. The protein expressions were quantified by ImageJ software.

## Results and Discussion

### Hypogen Pharmacophore Model

#### Construction of Hypogen Model

**FIGURE 2:**
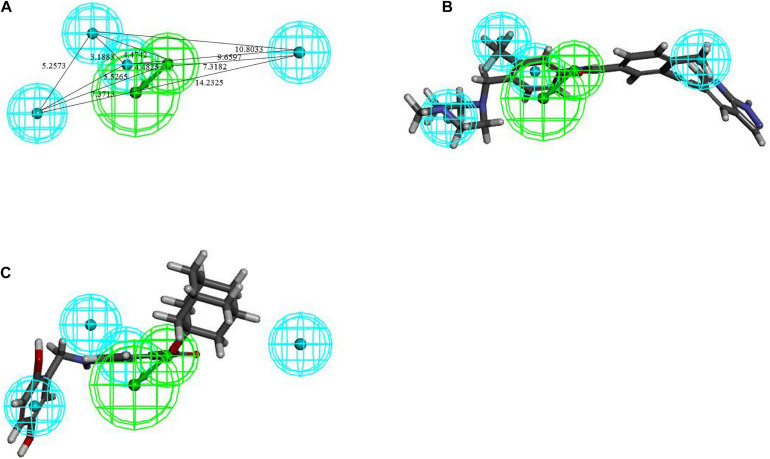
**(A)** 3D spatial relationship and geometric parameters of Hypogen1. **(B)** Hypogen1 mapping with the most active compound. **(C)** Hypogen 1 mapping with the least active compound.

The training set samples for the Hypogen modeling included 21 diverse BCR-ABL inhibitors obtained from different publications (Buchdunger et al., 1996; Nagar et al., 2002; Gumireddy et al., 2005; Barnes et al., 2007; Katsoulas et al., 2008; Azam et al., 2010; Ren et al., 2013; Bu et al., 2014; Pan et al., 2015; Zabriskie et al., 2015; Halbach et al., 2016; Salerno et al., 2017; Rossari et al., 2018) with diverse structures and broad efficacy values (IC_50_) that ranged from 0.0002 μM to 9.8 μM. The chemicals used to construct the Hypogen pharmacophore models are shown in Supplementary Figure 1. The top 10 pharmacophore models containing HBA, AR and H chemical features are shown in Supplementary Table 1. Among all of these models, Hypogen1, composed of HBA and 4H chemical features, had the lowest total cost (91.20), highest cost difference (69.50), lowest RMS (0.50), best correlation coefficient (0.99), and highest fit value (11.89). Cost analysis was applied to determine the statistical significance of the Hypogen models (Ye et al., 2010). The cost difference of Hypogen1 (69.4973), the difference between the null cost and total cost indicated more than 90 instances of statistical significance of achieving an acceptable pharmacophore (Kavitha et al., 2015; Kumar et al., 2015). The low RMS and high correlation coefficient suggested that the model is stable and internally predictive (Kandakatla and Ramakrishnan, 2014). The fit value was indicative of the overall fitness of the training set compounds on a pharmacophore model during pharmacophore generation (Dube et al., 2012). Therefore, the Hypogen1 was selected based on the highest correlation coefficient, lowest total cost and RMS value (Aparoy et al., 2010), 3D spatial relationship and distance constraints of Hypogen1 are shown in Figure 2A. The Figures 2B,C show the most active and inactive compounds in the training set aligned with Hypogen1.

If the compounds were divided into three categories based on their efficacies as being highly active (IC_50_ ≤ 0.1 μM, +++), moderately active (0.1 μM < IC_50_ < 3 μM, ++), and least active (IC_50_ ≥ 3 μM, +), this categorization could be used to rapidly estimate the predictive accuracy of the pharmacophore (Huang et al., 2012). Table 1 shows that the efficacies of the training set compounds could be accurately predicted.

**TABLE 1:** Actual and estimated efficacies of the training set molecules based on the Hypogen1 model.

Compound no.	Exp. IC_50_ μM	Predicted IC_50_ μM	Fit value	Error	Experimental scale	Predicted scale
1	0.0002	0.000276612	11.8863	1.38306	+++	+++
2	0.0005	0.000633086	11.5267	1.26617	+++	+++
3	0.007	0.013691	10.1918	1.95586	+++	+++
4	0.0071	0.0157362	10.1313	2.21637	+++	++
5	0.01	0.0144282	10.169	1.44282	+++	+++
6	0.019	0.0579635	9.56505	3.05071	+++	+++
7	0.025	0.0126119	10.2274	−1.98225	+++	+++
8	0.028	0.0287705	9.86925	1.02752	+++	+++
9	0.034	0.012146	10.2438	−2.79928	+++	+++
10	0.0495	0.0896702	9.37555	1.81152	+++	+++
11	0.0585	0.0555811	9.58327	−1.05252	+++	+++
12	0.2	0.194532	9.03921	−1.02811	++	++
13	0.8112	0.990206	8.33247	1.22067	++	++
14	2.59	2.31136	7.96433	−1.12055	++	++
15	2.9	3.51633	7.78211	1.21253	++	+
16	3.32	2.3407	7.95885	−1.41838	+	++
17	4.4	2.47184	7.93518	−1.78005	+	++
18	4.8	6.61342	7.50777	1.3778	+	+
19	7.02	4.71511	7.65471	−1.48883	+	+
20	7.5	3.51889	7.78179	−2.13135	+	+
21	9.8	4.26887	7.69789	−2.29569	+	+

#### Validation of Hypogen1

The test set, Fischer randomization and decoy set were used to verify the Hypogen pharmacophore model.

##### Test set prediction

**FIGURE 3:**
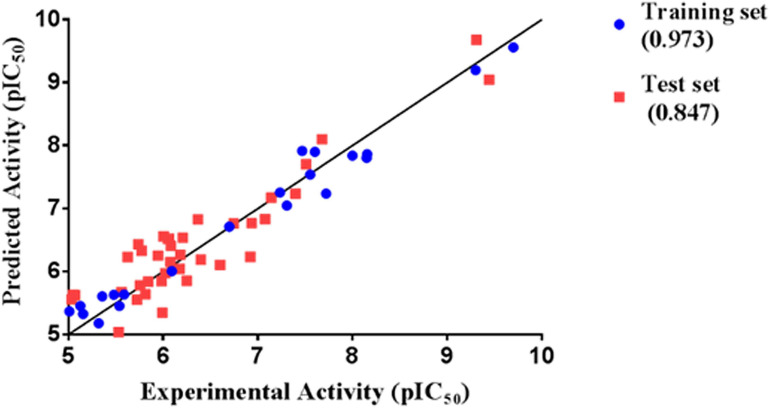
Correlation between the experimented and predicted efficacies of the training set and the test set based on the Hypogen1 model.

Thirty eight compounds, with different scaffold and efficacy ranges, were used as a test set (Supplementary Figure 2) to assess whether the pharmacophore model can accurately predict the efficacy of the compound (Huang et al., 2012). The experimental and predicted efficacies of the test set are shown in Table 2. A significant correlation coefficient of 0.847 was obtained for the test set compounds using regression analysis. The error factors of all compounds were less than 5%. Figure 3 shows the scatter plot of the experimental and the predicted efficacies, indicating that the samples are clustered near the diagonal line, y = x. These results indicated that the Hypogen1 model has excellent predictive validity.

**TABLE 2:** Actual and estimated efficacy of the test set molecules based on the Hypogen1 model.

Compound no.	Exp. IC_50_ μM	Predicted IC_50_ μM	Fit value	Error	Experimental scale	Predicted scale
1	0.00036	0.000896414	11.3757	2.49004	+++	+++
2	0.00049	0.000209471	12.0071	−2.33923	+++	+++
3	0.021	0.00797192	10.4266	−2.63425	+++	+++
4	0.031	0.019866	10.0301	−1.56046	+++	+++
5	0.04	0.0582672	9.56278	1.45668	+++	+++
6	0.072	0.0672314	9.50063	−1.07093	+++	+++
7	0.084	0.147936	9.15812	1.76117	+++	++
8	0.116	0.169831	9.09818	1.46406	++	++
9	0.12	0.584101	8.56171	4.86751	++	++
10	0.18	0.171855	9.09304	−1.04739	++	++
11	0.25	0.787807	8.43178	3.15123	++	++
12	0.4	0.647081	8.51724	1.6177	++	++
13	0.43	0.148644	9.15605	−2.89282	++	++
14	0.563	1.40413	8.18079	2.49401	++	++
15	0.62	0.291697	8.86327	−2.12549	++	++
16	0.66	0.543583	8.59293	−1.21417	++	++
17	0.67	0.902735	8.37264	1.34734	++	++
18	0.83	0.390716	8.73634	−2.12431	++	++
19	0.84	0.705346	8.4798	−1.1909	++	++
20	0.872	0.302548	8.84741	−2.88219	++	++
21	0.89	0.304132	8.84514	−2.92636	++	++
22	0.95	1.05843	8.30354	1.11414	++	++
23	0.99	0.278074	8.88404	−3.5602	++	++
24	1.02	4.48861	7.67609	4.4006	++	+
25	1.04	1.41841	8.1764	1.36386	++	++
26	1.13	0.560729	8.57945	−2.01523	++	++
27	1.44	1.44555	8.16817	1.00385	++	++
28	1.54	2.29464	7.96749	1.49003	++	++
29	1.69	0.466732	8.65913	−3.62092	++	++
30	1.76	1.66113	8.1078	−1.05952	++	++
31	1.83	0.370472	8.75944	−4.93964	++	++
32	1.89	2.79708	7.8815	1.47994	++	++
33	2.37	0.58676	8.55974	−4.03913	++	++
34	2.76	2.11978	8.00191	−1.30202	++	++
35	2.96	9.16837	7.36591	3.09742	++	+
36	8.53	2.35434	7.95633	−3.6231	+	++
37	8.95	2.32546	7.96169	−3.8487	+	++
38	9.25	2.76764	7.88609	−3.3422	+	++

##### Fischer randomization test

**FIGURE 4:**
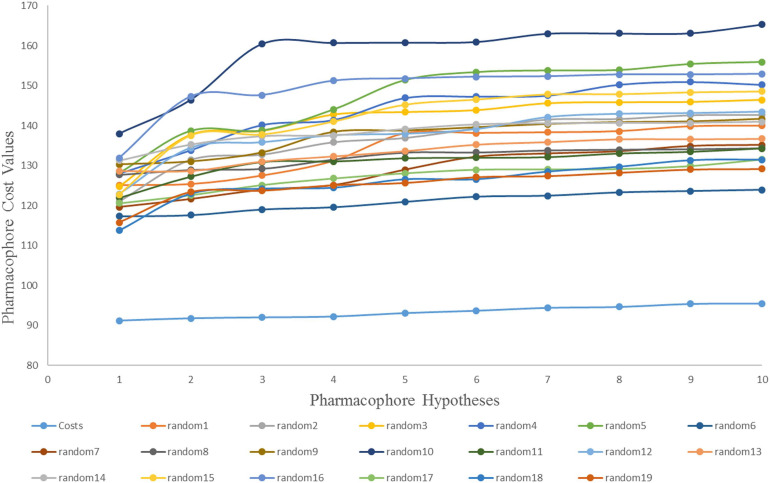
The difference in cost between the initial hypothesis and 19 random hypotheses. The 95% confidence level was selected to validate the Fischer randomization test results (Hypogen1).

The Fischer randomization test is a cross validation method that can be used to evaluate the statistical correlation between structures and activities in training set compounds (Pal et al., 2019). To achieve a confidence level of 95% for Fischer’s randomization, 19 random hypotheses were generated (Sakkiah et al., 2012). Figure 4 clearly shows that the Hypogen1 pharmacophore model was not generated by chance as the total cost of these randomly generated hypotheses is much higher than the initial hypothesis.

##### Decoy set

A decoy set was used to validate the efficiency of Hypogen1 by computing various parameters such as false positive, false negative, enrichment factor (EF), goodness of fit score (GF), total number of hit molecules (Ht), and total number of active compounds (Ha) (Halgren et al., 2004). The decoy set database (D) contained 325 compounds, including 25 BCR-ABL inhibitors and 300 inactive compounds. Our results indicated that 26 compounds were identified as being active based on the Hypogen1 model and 21 compounds were known inhibitors of the BCR-ABL protein. The relevant parameters for the decoy set are shown in Table 3. An EF of 10.5 and goodness of GF of 0.8022 verifies the high efficiency of Hypogen1, indicating the high predictive ability of the model.

**TABLE 3:** Statistical parameters of the decoy set for Hypogen1.

No	Parameter	Values
1	Total number of molecules in database (D)	325
2	Total number of actives in database (A)	25
3	Total number of hit molecules from the database (Ht)	26
4	Total number of active molecules in hit list (Ha)	21
5	% Yield of actives [(Ha/Ht) × 100]	80.77
6	% Ratio of actives [(Ha/A) × 100]	84
7	Enrichment factor^a^ (EF)	10.5
8	False negatives [A–Ha]	4
9	False Positives [Ht–Ha]	5
10	Goodness of fit score^b^ (GF)	0.8022

$$a\,\left(\frac{\text{Ha}\times \text{D}}{\text{Ht}\times \text{A}}\right)$$

$$b\,\left(\frac{H\,a}{4\,H\,t\,A}\right)\,\left(3\,A+H\,t\right)\times \left\{1-\left[\frac{H\,t-H\,a}{D-A}\right]\right\}$$

### Hiphop Pharmacophore Model

#### Construction of Hiphop Model

Eight compounds were selected as the training set to generate qualitative Hiphop models for common feature pharmacophore generation. The structures and bioactivities of the training set are shown in Supplementary Figure 3. The top 10 common feature hypotheses were produced and are shown in Supplementary Table 2, according to their ranking scores. Based on the ranking scores and feature similarities of hypotheses, ten hypotheses were clustered into two groups: the first group models contained 2RA, 3H, 1HBD, and 2HBA chemical features and the second group models contained 1RA, 4H, 1HBD, and 2HBA chemical features.

**FIGURE 5:**
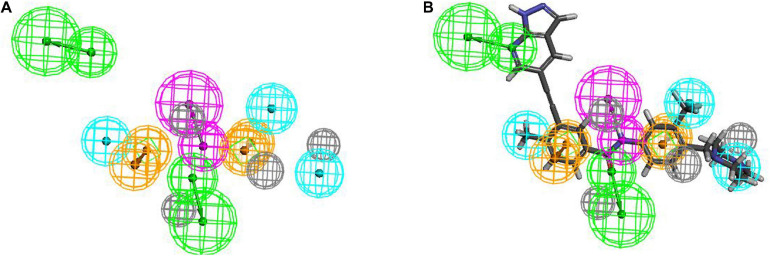
**(A)** The Hiphop1 pharmacophore model. **(B)** Hiphop1 mapped with the most active compound.

Hiphop1 and Hiphop9 were the top ranked models for each cluster and therefore, were selected for further analysis. The bestfit values of Hiphop1 and Hiphop9 model with all training set compounds are shown in Supplementary Table 3, and the average of the bestfit value for active and inactive compounds were calculated for each hypothesis. Based on the results in Supplementary Table 3, we can see that compared with Hiphop9, Hiphop1 has a more pronounced difference in the active and inactive compounds, and thus, Hiphop1 was chosen as the optimal Hiphop model (Figure 5A). Figure 5B shows the most active compound in the training set aligned with Hiphip1.

#### Validation of Hiphop1

To validate the Hiphop1 model, 21 active compounds, with different scaffold and efficacies (0.0005–41 μM) (Supplementary Figure 4) and 220 compounds with unknown efficacies from Maybridge, were combined to create a new database (D_1_). Hiphop1 was used to select the active compounds. As a result, 22 compounds were screened from the database by Hiphop1 and 21 of these compounds have been previously proven to be known effective compounds. The validation results (Table 4) indicated an EF of 10.95 and a goodness of GF of 0.91, thus validating the high efficiency of Hiphop1 and demonstrating that Hiphop1 can discriminate active compounds from inactive compounds.

**TABLE 4:** Statistical parameters of Hiphop-1.

No	Parameter	Values
1	Total number of molecules in database (D_1_)	241
2	Total number of actives in database (A)	21
3	Total number of hit molecules from the database (Ht)	22
4	Total number of active molecules in hit list (Ha)	21
5	% Yield of actives [(Ha/Ht) × 100]	95.45
6	% Ratio of actives [(Ha/A) × 100]	100
7	Enrichment Factor^a^ (EF)	10.95
8	False negatives (A–Ha)	0
9	False Positives (Ht–Ha)	1
10	Goodness of fit score^b^ (GF)	0.91

$$a\,\left(\frac{\text{Ha}\times \text{D}}{\text{Ht}\times \text{A}}\right)$$

$$b\,\left(\frac{H\,a}{4\,H\,t\,A}\right)\,\left(3\,A+H\,t\right)\times \left\{1-\left[\frac{H\,t-H\,a}{D-A}\right]\right\}$$

### Virtual Screening

The best validated Hypogen1 and Hiphop1 pharmacophore models were used as 3D queries to screen the drug-like subset of the ZINC Database. ZINC is a free database of purchasable molecules, including multiple subsets such as drug-like and lead-like and it offers similarity searching on its website (Irwin and Shoichet, 2005; Awale et al., 2015). The work flow for the virtual screening is shown in Figure 1. Overall, 31,966 compounds matched all of the chemical features of Hypogen1 and 4,188 compounds had fit values > 10. By using Hiphop1, 896 compounds were selected out for their high magnitude of matching with all of the chemical features of Hiphop and 192 compounds with fit values > 1.8 were selected. The hit compounds were further subjected to Lipinski’s rule of five and ADMET to assess their drug-like and pharmacokinetic properties (Rampogu et al., 2018), and 3,865 and 162 compounds meet these standards, respectively. Subsequently, molecular docking was used to determine if these compounds interact with the BCR-ABL protein. In total, 3,856 and 161 compounds were screened using Lipinski’s rule of five and ADMET, respectively, interacted with the active site of protein. Finally, identical compounds screened from the two pharmacophore models were selected for the *in vitro* efficacy assay. The docking scores for these compounds are shown in Supplementary Table 4.

### Compounds Inhibit the Proliferation of K562 Cells

There were 13 identical compounds (Supplementary Table 4) and we purchased six of these for the proliferation experiments: ZINC36617838, ZINC30201139, ZINC65008391, ZINC45895251, ZINC36617852, and ZINC36617849.

**FIGURE 6:**
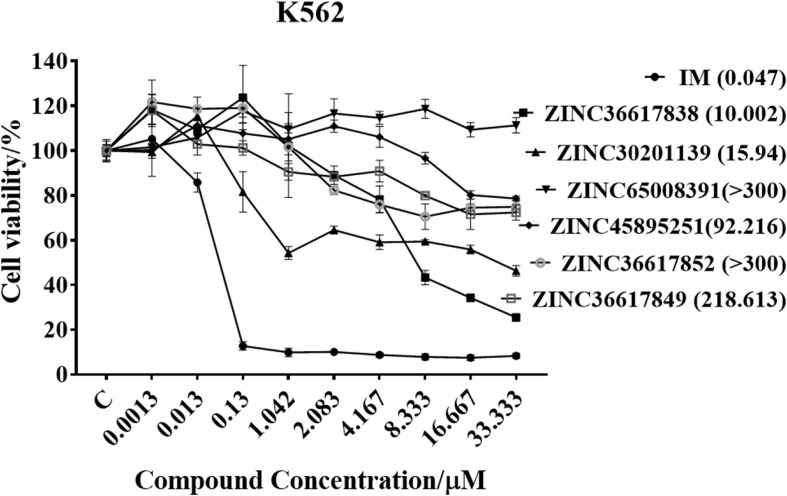
Cytotoxic efficacy of compounds in K562 leukemia cells. Cells were incubated with various concentrations of IM, ZINC36617838, ZINC30201139, ZINC65008391, ZINC45895251, ZINC36617852, or ZINC36617849 for 72 h. Cell viability was determined using the MTT assay. The results are represented as the mean ± SD.

MTT assays were performed to determine the inhibitory efficacy of compounds on the proliferation of K562 leukemia cells. The cells were incubated with various concentrations of the six compounds for 72 h and the results are shown in Figure 6. The compound, ZINC36617838, had a greater efficacy in inhibiting the proliferation of K562 leukemia cells (IC_50_ 10.002 μM) compared to the other ZINC compounds. However, the efficacy of ZINC36617838 was approximately 200 times lower than that of imatinib (IC_50_ 0.047 μM). This finding led us to select ZINC36617838 as a potential hit compound for structural optimization. The structure of ZINC36617838 and the interaction between ZINC36617838 and 1IEP is shown in Supplementary Figure 5.

### Structure Optimization

**FIGURE 7:**
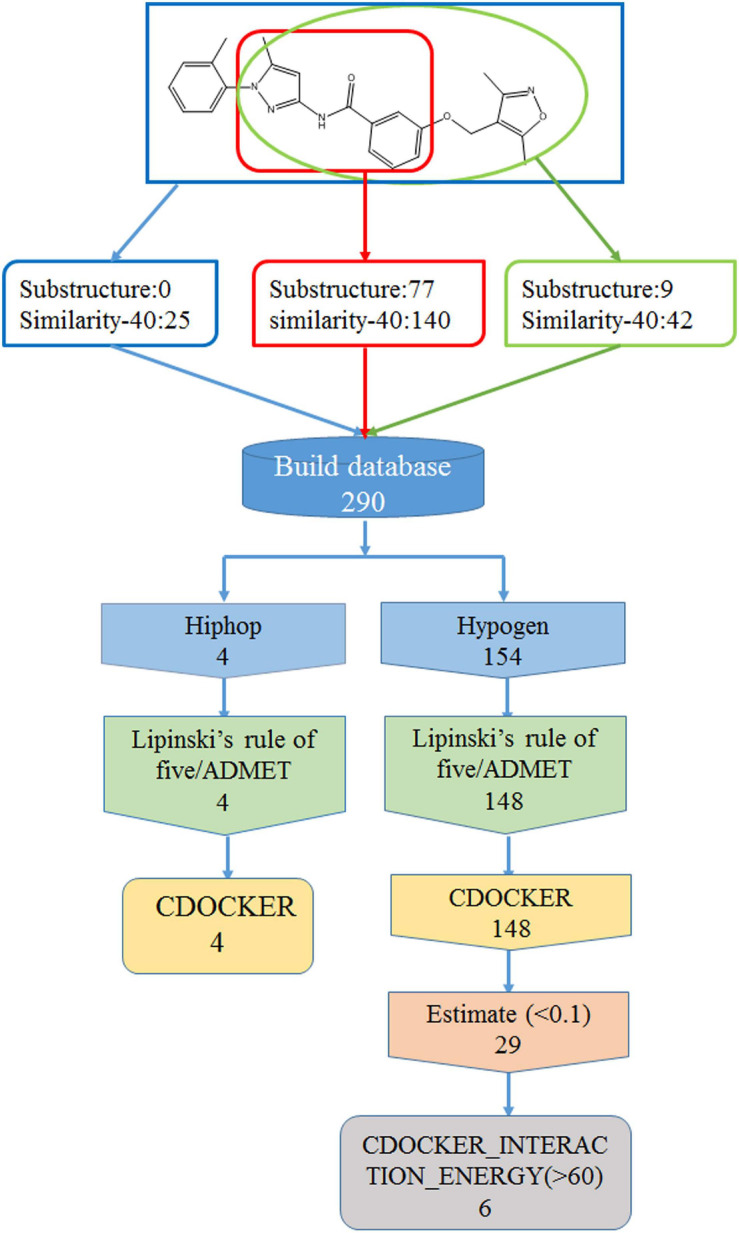
The work flow chart of structure optimization and its screening process.

In order to find new compounds with higher inhibitory efficacy, the skeleton structure of ZINC36617838 (Figure 7), was used as a substructure to search for its derivatives in the ZINC database. The compounds that had 40% similarity with ZINC36617838 were selected (circled in red, green, and blue in Figure 7). As shown in Figure 7, the analysis identified 290 compounds. The validated Hypogen1 and Hiphop1 pharmacophores were used as 3D queries to screen the database and four and 153 compounds matched all of the pharmacophore features of Hiphop1 and Hypogen1, respectively. Subsequently, four and 148 compounds meet the standards of the Lipinski’s rules of five and ADMET filters, respectively, and were docked into active site of protein to remove the detected false positives (Rampogu et al., 2018).

**FIGURE 8:**
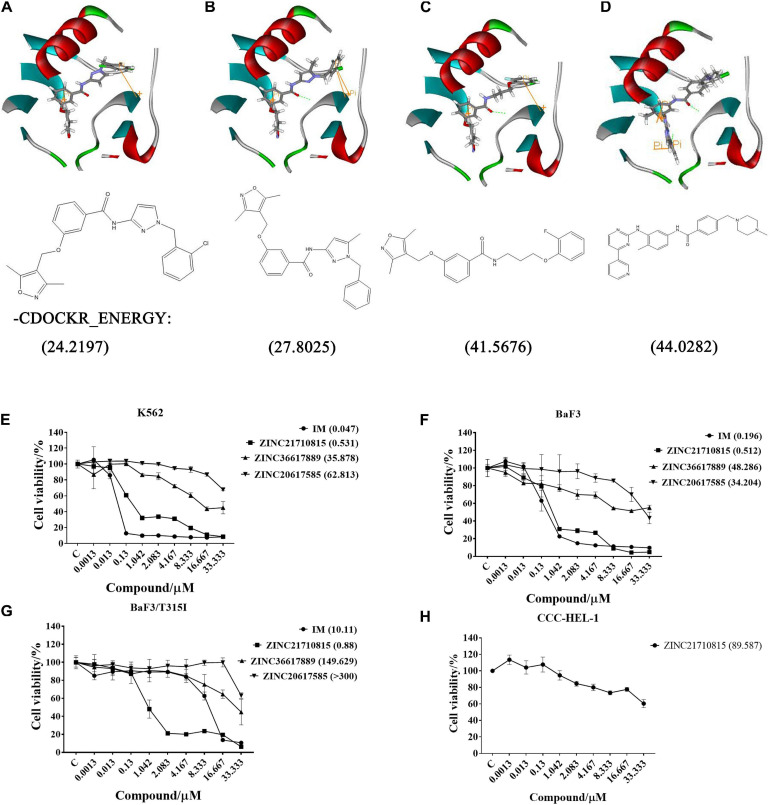
The binding modes of three compounds and imatinib with the BCR-ABL protein (PDB: 1IEP): hydrogen bond (green), pi-pi (yellow). **(A)** ZINC20617585; **(B)** ZINC36617889; **(C)** ZINC21710815; and **(D)** Imatinib (IM). The effect of these compounds on the proliferation of **(E)** K562, **(F)** BaF3, **(G)** BaF3/T315I leukemia cells, and **(H)** CCC-HEL-1 cells. The cells were incubated with various concentrations of imatinib (IM), ZINC21710815, ZINC36617889, or ZINC20617585 for 72 h. The viability of the cells was assessed using the MTT assay. The results are represented as the mean ± S.D.

After screening, four compounds from the Hiphop screening were also identified from the Hypogen screening. However, the predicted IC_50_ values or efficacy of 29 out of the 148 compounds were less than 0.1 μM in the Hypogen pharmacophore screening pathway, and the CDOCKER INTERACTION ENERGY of six compounds were greater than 60. Therefore, these ten compounds were selected for further experimental evaluation. We purchased ZINC21710815, ZINC36617889, and ZINC20617585. The binding modes of these three compounds are shown in Figures 8A–C.

We used the MTT assay to determine the efficacy of these compounds to inhibit the proliferation of K562, BaF3/WT, and BaF3/T315I leukemia cells (Figure 8). All cells were incubated with different concentrations of each compound for 72 h. As shown in Figure 8E, imatinib and ZINC21710815 inhibited the proliferation of K562 cells with IC_50_ values of 0.047 and 0.531 μM, respectively. The IC_50_ value for ZINC21710815 was approximately 10 times lower than that of imatinib. Imatinib and ZINC21710815 decreased the viability of BaF3/WT leukemia cells, with IC_50_ values of 0.196 and 0.512 μM, respectively (Figure 8F). In the BaF3/T315I leukemia cells, which have the T315I mutation that produces resistance to imatinib, ZINC21710815 significantly inhibited the proliferation of BaF3/T315I leukemia cells, (IC_50_ = 0.88 μM), and its IC_50_ value was significantly lower than that for imatinib (IC_50_ = 10.11 μM), and its IC_50_ for normal CCC-HEL-1 cells was 89.587 μM (Figure 8H). The selectivity indexes (SI) were 1, 168.71, 174.97, and 101.80, respectively, for the CCC-DEL-1, K562, BaF3/WT, and BaF3/T315I cells. These results suggest that ZINC21710815 was not cytotoxic in normal CCC-HEL-1 cells and it significantly inhibited the proliferation of wild-type BCR-ABL and T315I mutated BCR-ABL leukemia cells.

### ZINC21710815 Inhibits the Growth of CML Cells

**FIGURE 9:**
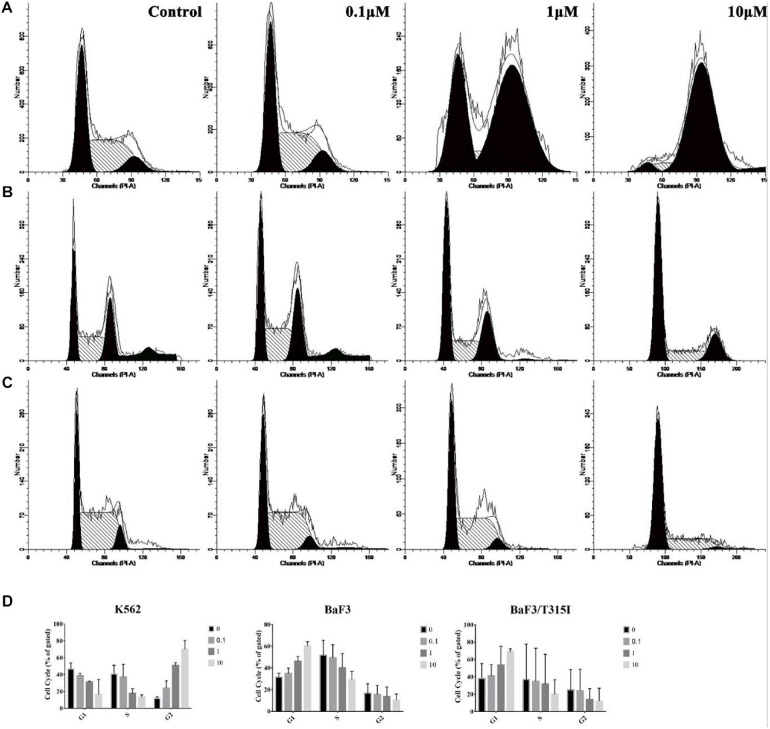
The effect of ZINC21710815 on the cell cycle in K562, BaF3/WT and BaF3/T315I leukemia cells. **(A)** K562 leukemia cells were incubated with various concentrations of ZINC21710815 for 24 h at a density of 1 × 10^6^ cells/well. **(B)** BaF3 leukemia cells were incubated with various concentrations of ZINC21710815 for 24 h at a density of 5 × 10^5^ cells/well. **(C)** BaF3/T315I leukemia cells were incubated with various concentrations of ZINC21710815 for 24 h at a density of 5 × 10^5^ cells/well. **(D)** The relative percentages of the cell cycles for K562, BaF3/WT, and BaF3/T315I leukemia cells were calculated using ModFit LT software. The results are represented as the mean ± SD.

Based on the proliferation experiments, we next determined the effect of ZINC21710815 on the cell cycle of the leukemia cells. The K562, BaF3/WT, and BaF3/T315I leukemia cells were incubated with various concentrations of ZINC21710815 for 24 h. These cells were incubated and stained with PI, which stains DNA, allowing for the analysis of cellular DNA content using flow cytometry. ZINC21710815 significantly increased the accumulation of K562 leukemia cells in the G_2_ phase, suggesting an arrest of the cells in the G_2_ phase (Figures 9A,D). In contrast, ZINC217108155 significantly increased the accumulation of BaF3/WT and BaF3/T315I cells leukemia cells in the G_1_ phase (Figures 9B,C,D). These findings suggested that ZINC21710815 may inhibit the proliferation of leukemia cells by interrupting the cell cycle in CML cells.

### ZINC 21710815 Induces Apoptosis in CML Cells

**FIGURE 10:**
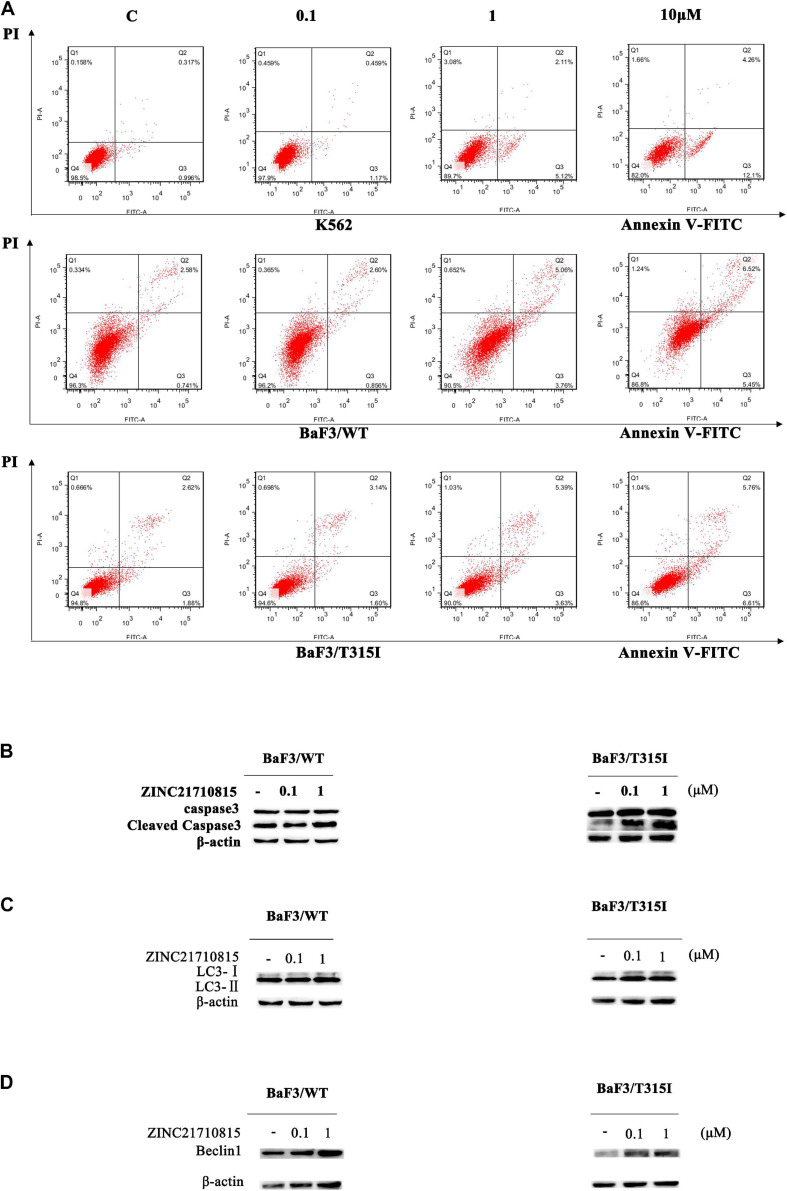
ZINC21710815 induces apoptosis and autophagy in CML cells. **(A)** K562, BaF3/WT, and BaF3/T315I cells were incubated with various concentration of ZINC21710815 for 24 h at a density of 5 × 10^5^ cells/well, stained using Annexin V-FITC and PT and analyzed by FlowJo. **(B)** Caspase-3 protein expression in BaF3/WT and BaF3/T315I cells incubated with 0 (vehicle), 0.1, or 1 μM of ZINC21710815 for 48 h. LC3 **(C)** and Beclin1 **(D)** protein expression in BaF3/WT and BaF3/T315I cells incubated with 0 (vehicle), 0.1, or 1 μM of ZINC21710815 for 48 h.

We conducted experiments to determine if ZINC21710815 inhibits the proliferation of K562, BaF3/WT, and BaF3/T315I leukemia cells by inducing apoptosis. The cells were cultured with various concentrations of ZINC21710815 for 24 h and the cells were stained using Annexin V-FITC/PI and analyzed using flow cytometry. As shown in Figure 10A, ZINC21710815 produce a concentration-dependent increase in the apoptosis of K562, BaF3/WT, and BaF3/T315I leukemia cells. Apoptosis is regulated by specific caspases, and cleaved caspase-3 has been shown to be a reliable marker for detecting apoptotic cells (Crowley and Waterhouse, 2016). Therefore, we measured the expression levels of caspase-3 using western blotting (Figure 10B). ZINC21710815 significantly increased the levels of cleaved caspase-3 in BaF3/WT and BaF3/T315I leukemia cells, suggesting that this compound induces apoptosis.

### ZINC21710815 Induces Autophagy in BaF3/WT and BaF3/T315I Leukemia Cells

To determine whether ZINC21710815 induced autophagy, we determined the expression of the autophagy-related proteins, LC3-II, and Beclin1. LC3-II is a hallmark protein of autophagy and the level of LC3-II expression is significantly correlated with the number of autophagosomes (Mizushima and Yoshimori, 2007; Yoshii and Mizushima, 2017; Lu et al., 2018). In the early stages of autophagosome formation, Beclin1 is considered to be an essential component for the initiation of autophagy (Hamurcu et al., 2018). As shown in Figures 10C,D, the level of LC3-II/LC3-I and Beclin1 are significantly increased in BaF3/WT and BaF3/T315I leukemia cells following incubation with ZINC21710815 for 48 h. These data suggest that ZINC21710815 induces autophagy, in part, by increasing the expression of the levels LC3-II and Beclin1 in BaF3/WT and BaF3/T315I leukemia cells.

### ZINC21710815 Inhibits Tyrosine Phosphorylation of the BCR-ABL Protein and Its Downstream Protein Targets, Signal Transducer and Activator of Transcription 5 (STAT5) and CRK Like Proto-Oncogene, Adaptor Protein (Crkl)

**FIGURE 11:**
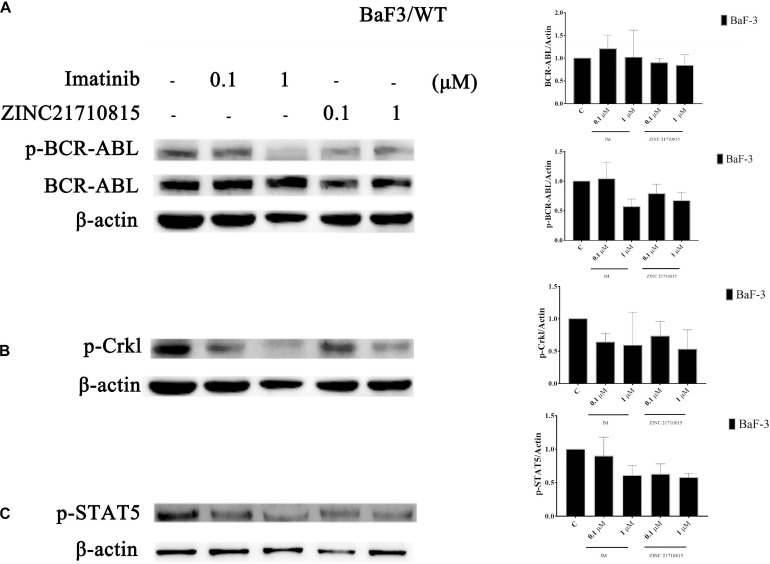
BaF3/WT leukemia cells were incubated with 0 (vehicle), 0.1, or 1 μM for 48 h with either imatinib and ZINC21710815 the levels of BCR-ABL and phosphorylated BCR-ABL **(A)**, and the downstream proteins p-Crkl **(B)**, p-STAT5 **(C)** were analyzed using western blotting. The results are represented as the mean ± SD.

BCR-ABL activates multiple downstream signaling pathways, including STAT5 and p-Crkl (Gupta et al., 2016). The activation of the transcription factor STAT5 is crucial for the progression of CML (Warsch et al., 2012). STAT5 was necessary for cellular proliferation and overexpression of constitutively active STAT5 could stimulate cell proliferation (de Groot et al., 1999). Crkl is an adaptor protein and a major substrate of BCR-ABL in CML cells (Oda et al., 1996). Crkl is phosphorylated by BCR-ABL and it subsequently activates other pathways that play a role in leukemic cell transformation (Senechal et al., 1996). To confirm the connection between the compound and BCR-ABL and downstream proteins, the effect of ZINC21710815 on the tyrosine phosphorylation of BCR-ABL and its downstream protein, STAT5 and Crkl, was determined using western blotting. BaF3/WT leukemia cells were incubated with various concentrations of imatinib and ZINC21710815 for 48 h. ZINC21710815 significantly reduced the phosphorylation of BCR-ABL at a concentration of 0.1 μM, decreased the phosphorylation of STAT5 at a concentration of 0.1 μM and reduced the phosphorylation of Crkl at a concentration of 1 μM in BaF3/WT leukemia cells (Figure 11).

## Discussion

In this study, Hypogen pharmacophore models were constructed based on 21 BCR-ABL TKIs and the best quantitative pharmacophore model consisted of five features. The correlation coefficient of Hypogen1 with the training and test set were 0.973 and 0.847, respectively. Hypogen1 was further validated by Fischer’s randomization method and by using a decoy set. Hiphop pharmacophore models were constructed based on five highly activity BCR-ABL inhibitors and three inhibitors with low efficacy. The best Hiphop pharmacophore model consists of eight features. The best pharmacophore models were selected to validate the test set and were highly efficient in distinguishing active from inactive compounds. The best Hypogen and Hiphop pharmacophore models were used as 3D queries to screen the ZINC database in order to find novel potential BCR-ABL inhibitors. The hit compounds were further screened by Lipinski’s rule of five, ADMET and molecular docking. Finally, three compounds, ZINC21710815, ZINC36617889, and ZINC20617585, were subjected to further analysis, of which compound ZINC21710815 had the greatest efficacy in inhibiting the proliferation of K562, BaF3/WT, and BaF3/T315I leukemia cells.

ZINC21710815, ZINC36617889, and ZINC20617585, as well as imatinib, interact with the BCR-ABL protein *via* a carbonyl of amide, which forms an O-NH hypogen interaction with Asp381, and all of these compounds form π-π interactions with residue Lys271. Given that imatinib binds to the ABL kinase domain known as the DFG-out conformation (Asp381-Phe382-Gly383) (Pereira et al., 2012), we hypothesize that ZINC21710815, ZINC36617889, and ZINC20617585 can induce the DFG-out conformation. Our results indicated that ZINC21710815 and imatinib have a higher –CDOCKER ENERGY compared to ZINC36617889 and ZINC 20617585 (Figure 8) and this could potentially explain why ZINC21710815 and imatinib have similar efficacies in inhibiting the proliferation of the leukemia cell lines used in this study. Imatinib inhibited the proliferation of K562 (IC_50_ = 0.047 μM), BaF3/WT (IC_50_ = 0.196 μM), and BaF3/T315I (IC_50_ = 10.11 μM) leukemia cells. ZINC21710815 was shown to be a novel compound that inhibited the proliferation of K562 (IC_50_ = 0.531 μM), BaF3/WT (IC_50_ = 0.512 μM), and BaF3/T315I leukemia cells (IC_50_ = 0.88 μM) in a concentration–dependent manner. ZINC21710815 and imatinib decreased the viability of K562 and BaF3/WT leukemia cells and ZINC21710815 produced a greater inhibition of BaF3/T315I leukemia cell viability compared to imatinib (Figure 8F). Although ZINC21710815 was the lead compound, it produced a lower magnitude of inhibition of BaF3/T315I leukemia cell proliferation compared to dasatinib in BaF3/WT and BaF3/T315T leukemia cells (IC_50_ = 1 nM/300 nM) and ponatinib in BaF3/WT and BaF3/T315I leukemia cells (IC_50_ = 1 nM/8 nM) (Cassuto et al., 2012), we will continue to optimize the structure of ZINC21710815 to improve its efficacy, particularly in the leukemia cells with the T315I mutation.

Our mechanistic experiments suggested that the ZINC21710815-induced decrease in the K562, BaF3/WT, and BaF3/T315I leukemia is due to it producing cell cycle arrest and apoptosis. ZINC21710815 increased the accumulation of K562 leukemia cells the G_2_ phase in and increased accumulation of BaF3/WT and BaF3/T315I leukemia cells in the G_1_ phase. As reported, imatinib induced an accumulation in the S phase, and dasatinib caused accumulation at the G_1_ phase and were efficacious in inhibiting leukemia cell proliferation (Song et al., 2013; Wu et al., 2014). In contrast, ZINC21710815 significantly increased the accumulation of cells in the G_1_ or G_2_ phases. These results suggest that ZINC21710815, in part, decreases the proliferation of the leukemia cell lines used in this study by producing cell cycle arrest in either the of the G_1_ or G_2_ phases. The anti-proliferative efficacy of ZINC21710815 may also be due to it inducing the apoptosis of CML cells as this compound increased the expression of the apoptosis-inducing protein, cleaved capase-3 in BaF3/WT and BaF3/T315I. It has been reported that the activation of the enzyme caspase-3 plays a critical role in inducing cancer cell apoptosis, and imatinib, ponatinib, and dasatinib can increase the levels of the caspase-3 by increasing the proteolysis of its inactive zymogen, pro-caspase-3, thereby increasing apoptosis (Quintás-Cardama et al., 2008; Okabe et al., 2014; Wu et al., 2014). Similarly, ZINC21710815, at 0.1 uM, increased the level of cleaved caspase-3, producing leukemia cell apoptosis. Finally, our results indicated that ZINC21710815 induces autophagy in BaF3/WT and BaF3/T315I leukemia cells, based on the increase in the expression of the autophagy-related proteins LC3-II and Beclin1, in BaF3/WT, and BaF3/T315I leukemia cells. Numerous studies have reported that the induction of autophagy by various TKIs, including imatinib and dasatinib, may protect CML cells from death, thereby promoting their survival (Bellodi et al., 2009; Helgason et al., 2011). Indeed, the inhibition of the BCR-ABL kinase activity is a main trigger of autophagy that allows the cells to survive in a stressful environment (Bellodi et al., 2009; Calabretta and Salomoni, 2012). *In vitro* studies indicate that the inhibition of autophagy increases the efficacy of certain TKIs in CML cells (Bellodi et al., 2009; Salomoni and Calabretta, 2009; Crowley et al., 2011; Yu et al., 2012). In the current study, we do not know if ZINC21710815’s induction of autophagy increases or decreases its anti-proliferative efficacy. Therefore, future experiments, where autophagy of the three leukemia cell lines is inhibited, must be done to ascertain if ZINC21710815’s efficacy is increased or decreased. However, it is important to note that the induction of autophagy in leukemia cells by imatinib increases the sequestration of the BCR-ABL protein in autophagosomes, decreasing its levels, thereby attenuating its oncogenic efficacy (Elzinga et al., 2013). Furthermore, the inhibition of autophagy in wild type and T315I leukemia cells decreases the biodegradation of the BCR-ABL protein, thus increasing its stability (Shinohara et al., 2019).

In order to further determine the efficacy ZINC21710815 on the BCR-ABL kinase, we measured the effect of ZINC21710815 on levels of the proteins STAT5 and Crkl, which are substrates for the BCR-ABL kinase. BCR-ABL kinase phosphorylates and activates the protein transcription factor, STAT5, which is translocated to the nucleus, where it increases the transcription of genes that produce proteins that promote cell survival and proliferation (Huang et al., 2002). Our results indicated that ZINC21710815 and imatinib, at 0.1 μM, significantly decreased p-STAT5 level in BaF3/WT leukemia cells. Our imatinib results are congruent with previous studies in leukemia cells (Dong et al., 2018; Tu et al., 2018) and similarly, dasitinib also decreases p-STAT5 levels in BaF3/WT leukemia cells (Fiskus et al., 2006). We also determined the effect of ZINC21710815 on the levels of phosphorylated Crkl, a protein that is phosphorylated by the BCR-ABL (Bhat et al., 1997). Phosphorylated Crkl is one of main tyrosyl-phosphoproteins present in peripheral blood cells of CML patients, where it functions as a nuclear adaptor and transcriptional activator in BCR-ABL expressing cells (Nichols et al., 1994; Rhodes et al., 2000). Furthermore, the levels of phosphorylated Crkl can be used as an indirect marker of BCR-ABL function and its levels can be used to determine the status of BCR-ABL kinase activity due to the fact that Crkl is only phosphorylated by BCR-ABL (Nichols et al., 1994; Hamilton et al., 2006). Our *in vitro* results indicated ZINC21710815 significantly decreased the levels of phosphorylated Crkl in BaF3/WT leukemia cell lines, the magnitude of inhibition of phosphorylated Crkl levels by ZINC21710815 was similar to that of imatinib. Similarly, dasatinib has been reported to decrease p-Crkl (Fiskus et al., 2006) levels with an efficacy comparable to that of ZINC21710815. Based on these findings, we hypothesize that ZINC21710815 may decrease leukemia cell proliferation by decreasing the phosphorylation of BCR-ABL, STAT5, and Crkl.

Our experimental results showed that the compound ZINC21710815 inhibits the proliferation of leukemia cells, inhibits cell cycle, induces autophagy and apoptosis and inhibits the tyrosine phosphorylation of BCR-ABL target protein and downstream proteins. However, ZINC21710815 had no significant effect on the expression of BCR-ABL kinase in the T315I leukemia cells. Therefore, in the future, we will conduct experiments to optimize the structure of ZINC21710815 to increase its inhibitory efficacy in T315I leukemia cells.

The BCR-ABL fusion gene can be seen in CML patients, it is also found in other types of leukemia patients, such as acute myeloid leukemia (AML) patients (Bucur et al., 2013), B-cell acute lymphoblastic leukemia (B-ALL) patients (Zhou et al., 2018), and mixed phenotype acute leukemia (MPAL) patients (Alexander et al., 2018). ZINC21710815 could decrease the expression of BCR-ABL, which may indicate that the compound may also have an inhibitory effect in other cancers.

In conclusion, ZINC21710815 significantly decreased the proliferation of K562, BaF3/WT, and BaF3/T315I leukemia cells due to it (1) producing cell cycle arrest; (2) inducing apoptosis; and (3) inhibiting the phosphorylation of BCR-ABL kinase and the downstream targets, STAT5 and Crkl, in BaF3/WT leukemia cells. As stated above, it remains to be determined if inducing autophagy increases or decreases the anti-proliferative efficacy of ZINC21710815. Overall, our results suggest that ZINC21710815 is an inhibitor of BCR-ABL tyrosine kinase activity. Additional studies must be done to determine the *in vivo* efficacy and toxicity of ZINC21710815.

## Data Availability Statement

The original contributions presented in the study are included in the article/Supplementary Material, further inquiries can be directed to the corresponding author/s.

## Author Contributions

T-TH and XW designed research. T-TH, S-JQ, and Z-XW performed research. T-TH and Z-NZ analyzed data. T-TH and CA wrote the manuscript. CA and Z-SC revised the manuscript. J-ZL and Z-SC supervised the research. All authors contributed to the article and approved the submitted version.

## Conflict of Interest

The authors declare that the research was conducted in the absence of any commercial or financial relationships that could be construed as a potential conflict of interest.
