# Supplementary material for: The Discovery of Novel BCR-ABL Tyrosine Kinase Inhibitors Using a Pharmacophore Modeling and Virtual Screening Approach
Source: Front Cell Dev Biol. 2021 Mar 4;9:649434. doi: 10.3389/fcell.2021.649434 (PMC7969810; doi:10.3389/fcell.2021.649434)
Supplement: Supplementary file 1 [file Data_Sheet_1.PDF]

Supplementary Material

Figure S1. Chemical structures of the training set and their IC_{50} values in parentheses (Hypogen)

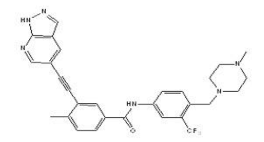

1 (0.0002μM)

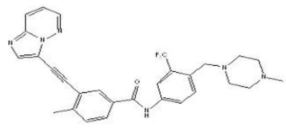

2 (0.0005μM)

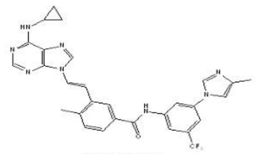

3 (0.007μM)

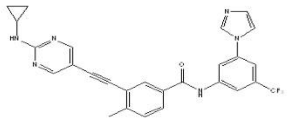

4 (0.0071μM)

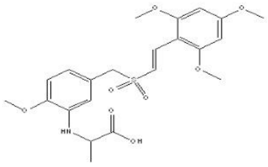

5 (0.01μM)

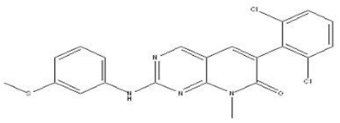

6 (0.019μM)

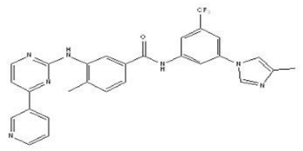

7 (0.025μM)

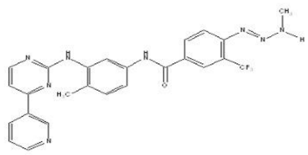

8 (0.028μM)

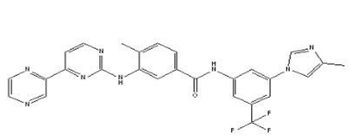

9 (0.034μM)

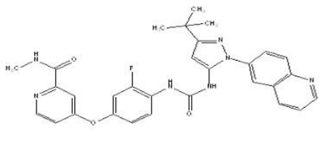

10 (0.0495μM)

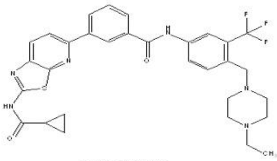

11 (0.0585μM)

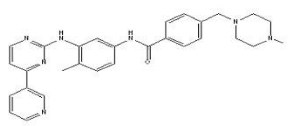

12 (0.2μM)

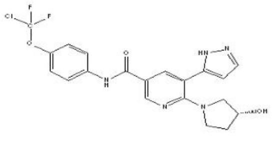

13 (0.8112μM)

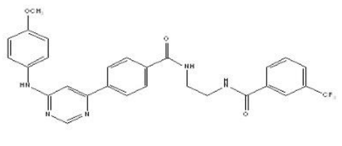

14 (2.59μM)

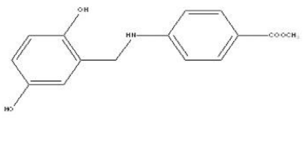

15 (2.9μM)

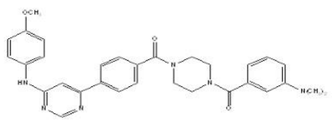

16 (3.32μM)

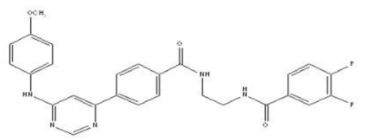

17 (4.4μM)

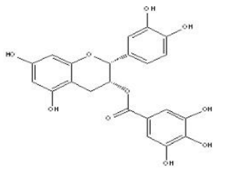

18 (4.8μM)

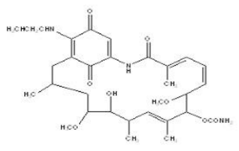

19 (7.02μM)

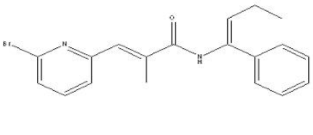

20 (7.5μM)

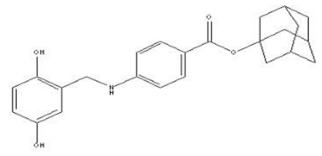

21 (9.8μM)

Figure S2. Chemical structures and their IC_{50} values in parentheses (Hypogen)

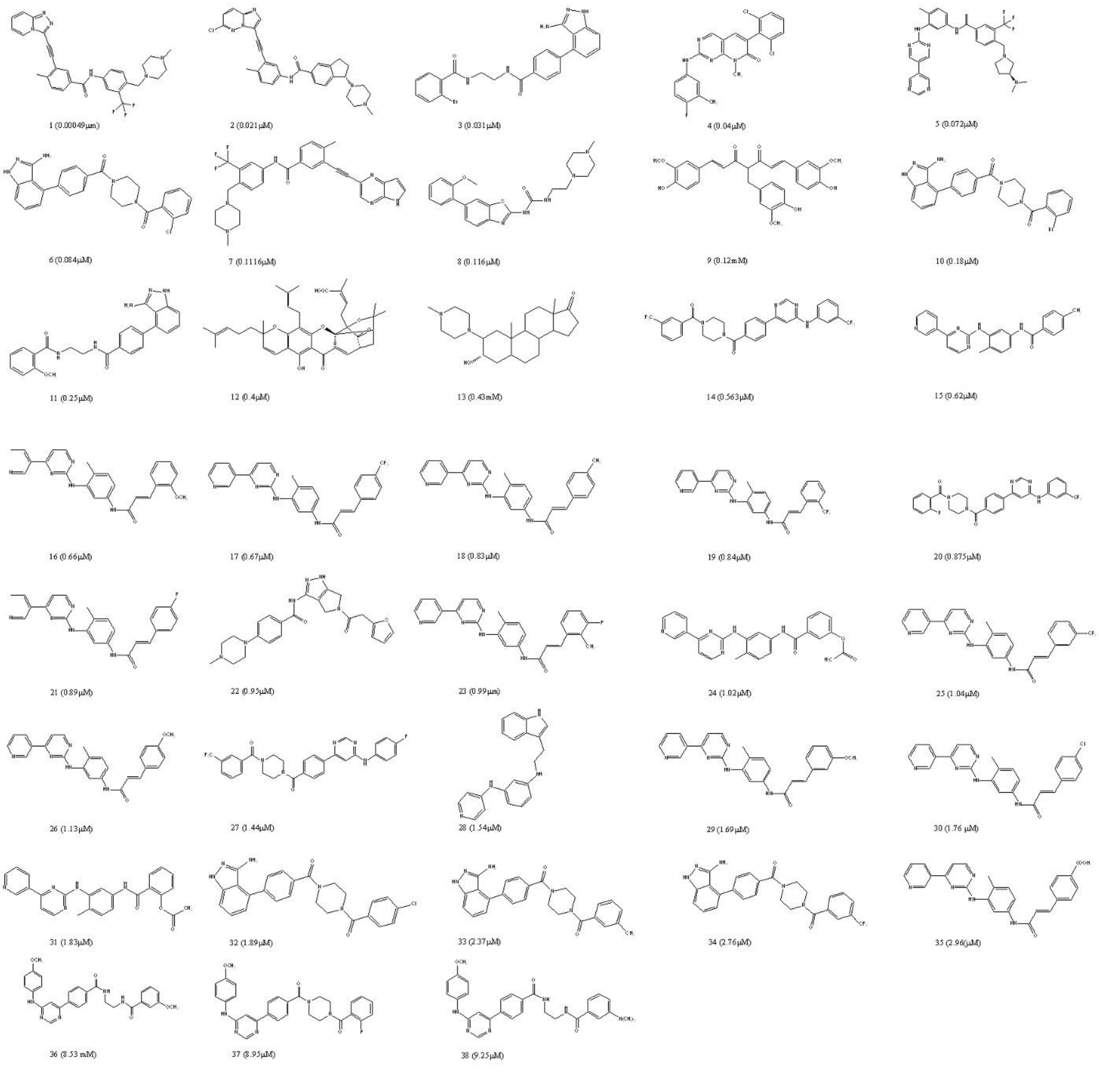

Figure S3. Chemical structures of the training set and their IC_{50} values in parentheses (Hiphop)

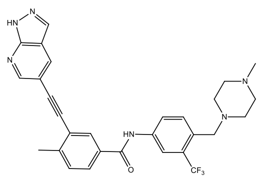

1 (0.0002μM)

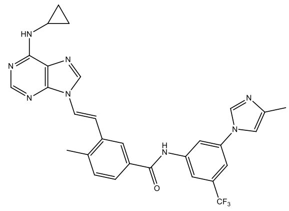

2 (0.007μM)

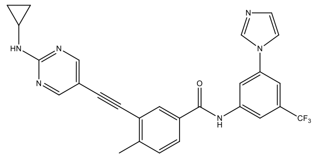

3 (0.0071μM)

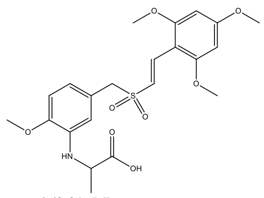

4 (0.01μM)

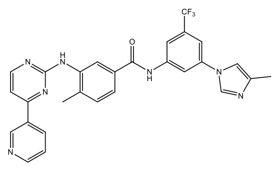

5 (0.025μM)

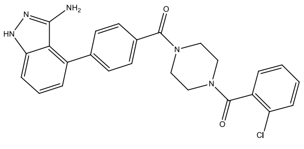

11M(8.56μM)

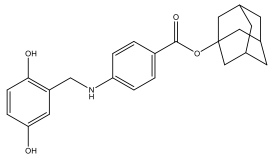

NSC680410(9.8μM)

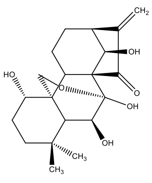

Oridonin(14.6μM)

Figure S4. Chemical structures and their IC_{50} values in parentheses (Hiphop) for the test set.

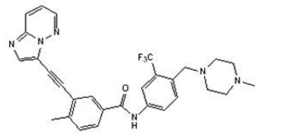

1 (0.0005μM)

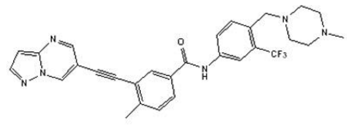

2 (0.0199μM)

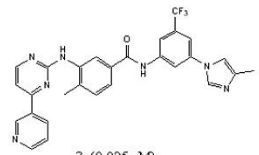

3 (0.025μM)

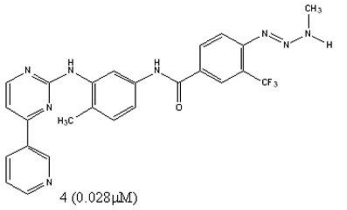

4 (0.028μM)

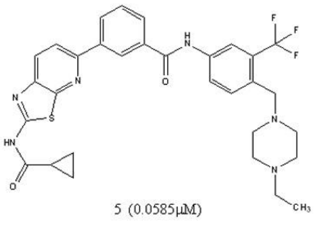

5 (0.0585μM)

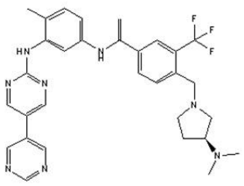

6 (0.072μM)

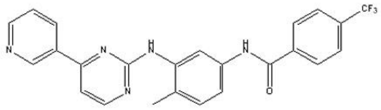

7 (0.59μM)

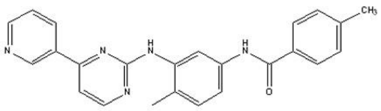

8 (0.62μM)

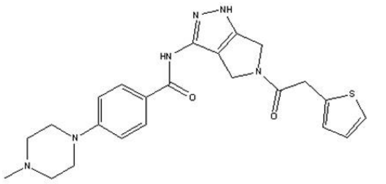

9 (0.95μM)

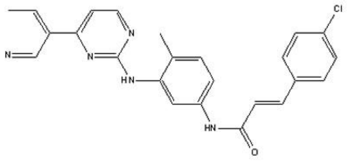

10 (1.76μM)

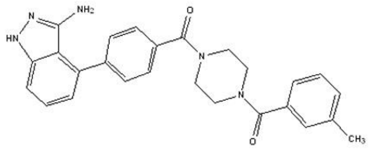

11 (2.37μM)

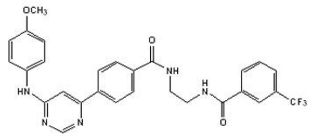

12 (2.59μM)

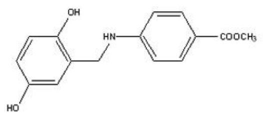

13 (2.9μM)

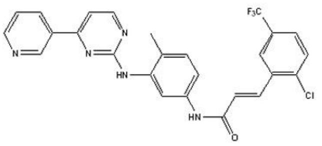

14 (4.13μM)

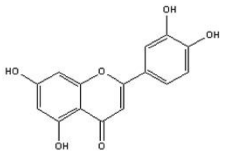

15 (5.4μM)

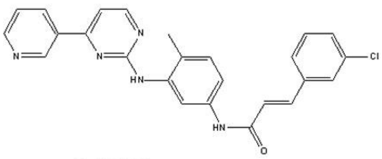

16 (5.41μM)

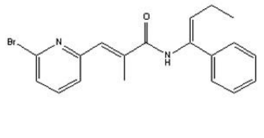

17 (7.5μM)

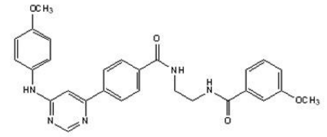

18 (8.53μM)

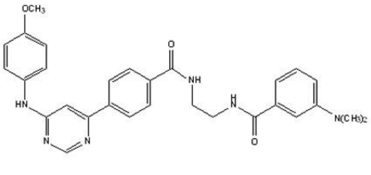

19 (9.25μM)

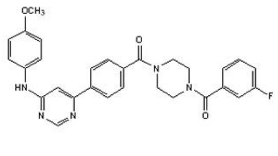

20 (23.51μM)

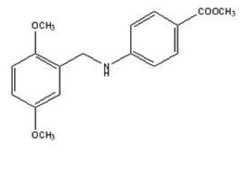

21 (41μM)

Figure S5. The ligand interaction diagram illustrating the interaction of ZINC36617838 and 1IEP: hydrogen bond (green), pi-pi (yellow)

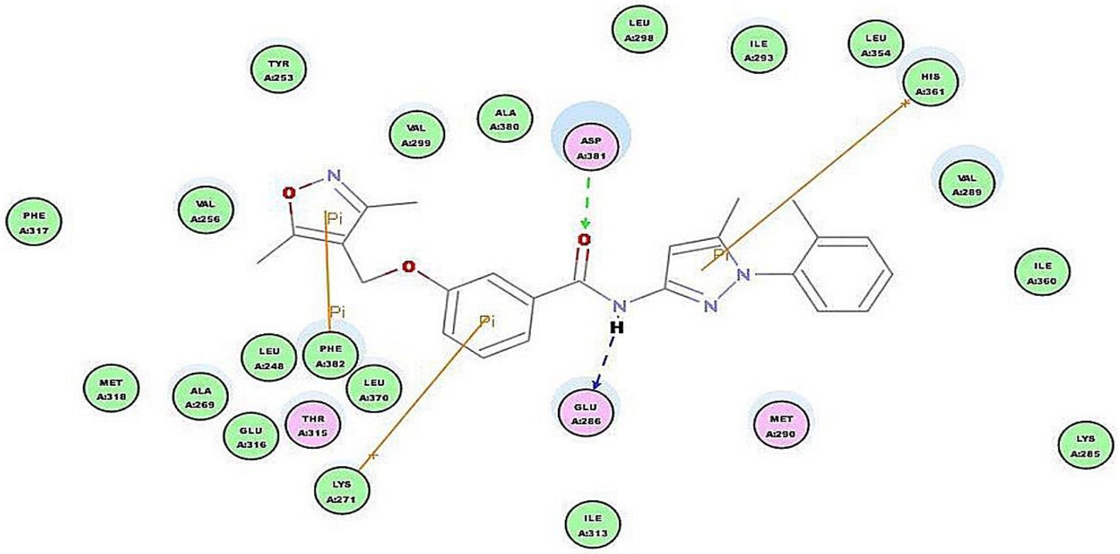

Table S1. Statistical parameters of the top 10 Hypogen pharmacophores models

Hypo No.	Total Cost	Cost difference[a]	RMS	correlation	Features[b]	Max. fit
1	91.20	69.50	0.50	0.99	HBA、H、H、H、H	11.89
2	91.78	68.92	0.67	0.97	HBA、H、H、H、H	11.04
3	92.01	68.69	0.63	0.98	HBA、HA、H、H、H	11.60
4	92.21	68.48	0.72	0.97	HBA、HA、H、H、H	10.89
5	93.08	67.62	0.69	0.97	HBA、HA、HA、H、H	11.81
6	93.65	67.04	0.72	0.97	HBA、HA、H、H、H	11.67
7	94.38	66.31	0.78	0.96	HBA、HA、H、H、H	11.66
8	94.61	66.09	0.89	0.95	HBA、H、H、H、H	9.95
9	95.36	65.33	0.92	0.95	HBA、HA、H、H、H	10.49
10	95.48	65.22	0.92	0.95	HBA、H、H、H、H	10.81

[a] The cost difference is the difference between the null cost and total cost. (The null cost, the fixed cost and the configuration cost are 160.695, 86.169 and 14.408 bits, respectively.)

[b] HBA, hydrogen bond donor; HA, hydrophobic_aromatic; H, hydrophobic;

[b] HBA, hydrogen bond donor; HA, hydrophobic_aromatic; H, hydrophobic;

Table S2. Details of the top ten hypotheses generated using Hiphop.

Hypothesis	Features[a]	Ranking score[b]	Direct hit (DH)[c]	Partial hit (PH)[d]	Max Fit	Cluster
Hiphop1	RA、RA、H、H、H、HBD、HBA、HBA	103.546	10111	01000	8	I
Hiphop2	RA、RA、H、H、H、HBD、HBA、HBA	103.302	10111	01000	8	I
Hiphop3	RA、RA、H、H、H、HBD、HBA、HBA	103.267	10111	01000	8	I
Hiphop4	RA、RA、H、H、H、HBD、HBA、HBA	103.267	10111	01000	8	I
Hiphop5	RA、RA、H、H、H、HBD、HBA、HBA	103.039	10111	01000	8	I
Hiphop6	RA、RA、H、H、H、HBD、HBA、HBA	102.864	10111	01000	8	I
Hiphop7	RA、RA、H、H、H、HBD、HBA、HBA	102.864	10111	01000	8	I
Hiphop8	RA、RA、H、H、H、HBD、HBA、HBA	102.748	10111	01000	8	I
Hiphop9	RA、H、H、H、H、HBD、HBA、HBA	99.808	10111	01000	8	II
Hiphop10	RA、H、H、H、H、HBD、HBA、HBA	99.808	10111	01000	8	II

[a] Features: RA: ring_ aromatic, H: hydrophobic, HBD: hydrogen bond donor, HBA: hydrogen bond acceptor

[b] Higher ranking score corresponds to a lower possibility of mapping to an inactive compound.

[c] Direct hit indicates whether (1) or not (0) a training set compound was mapped to every feature in the hypothesis.

[d] Partial hit indicates wheather (1) or not (0) a training set compound was mapped to all but one feature in the hypothesis.

Table S3. Biological data and estimated BestFit values of HipHop training set molecules based on the top-ranked models in each cluster.

Compound	Act (μM)	BestFit value		BestFit value average	
		Hiphop1	Hiphop9	Hiphop1	Hiphop9
GZD824	0.0002	7.99931	4.49304	4.498194	3.191322
AP24163	0.007	4.53456	3.89206		
S116836	0.0071	3.76738	1.49993		
ONO12380	0.01	1.81166	1.58654		
Nilotinib	0.025	4.37803	4.48504		
11M	8.56	1.72006	3.03154	1.242337	2.64991
NSC68040	9.8	1.40264	2.77518		
Oridonin	14.6	0.60431	2.14301		

Table S4. CDOCKER docking scores of 13 ZINC compounds.

N0	compounds	-CDOCKER_ENERGY	-CDOCKER_INTERACTION_ENERGY
1	ZINC30529452	43.6082	63.6025
2	ZINC79414029	35.4026	48.2234
3	ZINC30201139	35.1917	55.5719
4	ZINC20142376	35.1755	63.127
5	ZINC36617838	32.3777	65.638
6	ZINC36617849	31.1327	67.5621
7	ZINC36617852	30.3903	64.0732
8	ZINC65008391	29.5365	55.5454
9	ZINC93018879	26.2588	59.8855
10	ZINC56719678	24.6669	59.478
11	ZINC92212196	-1.27587	53.3983
12	ZINC12327273	-7.23305	62.1283
13	ZINC45895251	-18.6864	55.0781
